# Does Calm Always Follow the Storm? A Comprehensive Temporal Analysis of Emergency Department Visits in Northern Italy Before and After the COVID-19 Pandemic

**DOI:** 10.3390/epidemiologia6010010

**Published:** 2025-03-01

**Authors:** Maria José De la Rosa, Andrea Duca, Lorenzo Querci, Francesca Cortellaro, Martina Calderaro, Paolo Pausilli, Annalisa Bodina, Andrea Albonico, Gabriele Perotti, Carlo Signorelli, Massimo Lombardo

**Affiliations:** 1 Faculty of Medicine, School of Public Health, Università Vita-Salute San Raffaele, 20132 Milan, Italy; 2 Agenzia Regionale Emergenza Urgenza, 20124 Milan, Italy; a.duca@areu.lombardia.it (A.D.);; 3 ASST Grande Ospedale Metropolitano Niguarda, 20162 Milan, Italy; 4 ASTIR Group, 20124 Milan, Italy

**Keywords:** emergency service, hospital, emergency room visits, crowding, COVID-19

## Abstract

Background/Objectives: Emergency department (ED) crowding has become a pressing global concern exacerbated by the COVID-19 pandemic. No studies have addressed this issue in Europe during the post-pandemic period so far. This study examined ED visit volumes, patient acuity, hospital admission rates, emergency vehicle arrivals, and crowding metrics before, during, and after the pandemic. Methods: We conducted a retrospective descriptive study including data on all ED visits in the Lombardy Region of Italy from January 2019 to December 2023. Furthermore, an inferential statistical analysis was performed to compare ED trends between 2019 and 2023. Results: During the analyzed period, there were 15,515,128 visits across all Lombardy EDs. ED visits dropped from 3,514,426 in 2019 to 2,380,005 in 2020, then rebounded to 3,464,756 in 2023. In 2019, triage code distribution was 9.9% white, 68.7% green, 19.0% yellow, and 1.9% red. During the pandemic, the proportion of white and green codes decreased. By 2023, these comprised 80.7% of the total. The percentage of admitted patients was 11.9% in 2019, rose to 16.2% in 2020, and returned to 11.4% in 2023. The median ED length of stay (EDLOS) for admitted patients in 2023 was 5.2 h (IQR [2.1–17.4]), compared to 3.8 h (IQR [1.6–8.6]) in 2019 (*p*-value < 0.01). The median EDLOS for discharged patients in 2023 was 2.7 h (IQR [1.4–4.9]), compared to 2.4 h (IQR [1.3–4.4]) in 2019 (*p*-value < 0.01). The rate of patients leaving before completing treatment was 5.0% in 2019 and peaked at 6.8% in 2023 (*p*-value < 0.01). Conclusions: In 2023, ED visits in Lombardy increased, compared to the pandemic period, but remained below 2019 levels. The proportion of high-acuity codes and hospital admissions was slightly lower than in 2019. However, ED crowding metrics worsened. The high levels of lower-acuity visits and the deterioration in crowding metrics highlight systemic challenges within the healthcare system.

## 1. Introduction

Emergency department (ED) crowding is a significant global issue exacerbated by the COVID-19 pandemic [1,2,3,4]. It is considered a sentinel indicator of health system performance. While frequently attributed solely to ED operations and inefficiencies, it reveals a broader health system dysfunction. In addition to its conventional role of providing immediate and critical medical assistance, the emergency care system has evolved into a vital source of primary healthcare for individuals without insurance or with limited access to community services. This expansion in function has led to increased congestion within EDs [5,6,7]. Another concern is the increasing number of visits from patients with complex and chronic conditions, especially among the elderly [8]. Compared to other age groups, older adults have a higher rate of ED utilization, longer stays, and greater need for resources and medical interventions [9]. Despite these considerations, inpatient access block has been identified as the primary cause of crowding [8,10,11,12]. It refers to a situation where patients cannot access suitable hospital beds within a reasonable timeframe [13]. This phenomenon is facilitated by low inpatient bed capacity, shortages in healthcare personnel, and insufficient post-acute care facilities [2,3,5]. According to a 2022 Italian national deliberation, the ED length of stay (EDLOS) of patients who need to be admitted should not exceed 8 h [14]. Managing patients awaiting hospital beds considerably increases the ED’s workload [15]. The consequences of ED crowding on patient safety and medical staff have been extensively described but are often underestimated. During periods of crowding, patients experience delays in assessment and necessary care, heightened exposure to errors, decreased satisfaction, prolonged inpatient stays, and increased mortality rates [5,8,10,16]. Staff endure heightened stress, greater exposure to violence, and a tendency to deviate from best practice guidelines [5,8]. Before the COVID-19 pandemic, numerous countries, including Italy, struggled with strained emergency services [5,6,17,18,19]. A study by Pines et al. across 15 countries highlighted that most showed both objective increases in ED visit rates and crowding [17]. The pandemic further intensified factors associated with ED crowding, leading to increased patient EDLOS [20]. ED visit volume declined dramatically in the early pandemic [1,21,22]. This decline was attributed to several factors, such as fear of contracting COVID-19 and fewer accidents and injuries due to isolation measures [23,24,25,26,27]. A study conducted in the United States of America analyzed ED visit volume and crowding measures from March 2020 to August 2022 across 18 states. The study revealed that since the beginning of the pandemic, visit volumes have not returned to 2019 levels; however, the EDLOS, door-to-clinician times, and rates of leaving without treatment were higher than those observed before the pandemic. This was attributed to unprecedented nurse shortages affecting both EDs and hospitals and shortages in other positions like technicians and laboratory personnel, which worsened the situation [1]. The healthcare system in Italy is characterized by universal coverage and is primarily funded through general taxation. It is decentralized into 20 regional health systems. Lombardy, the most populous region in Italy, is served by the Lombardy Health Service, providing healthcare to approximately 10 million residents through a network of state and affiliated private hospitals. The emergency hospital network consists of facilities of varying levels of complexity, interconnected through the integrated hub-and-spoke model, with roughly one ED per 100,000 inhabitants. This study evaluates the impact of the COVID-19 pandemic on ED visits and crowding metrics in Lombardy, Italy, focusing on their evolution in the post-pandemic period. It aims to assess whether crowding metrics worsened in 2023 compared to 2019. ED volume, patient acuity, hospital admissions, and crowding measures were analyzed across three periods: pre-pandemic (2019), pandemic (2020–2022), and post-pandemic (2023). The analysis will provide valuable insights into the effects of the pandemic on ED operations and highlight ongoing challenges within the healthcare system.

## 2. Materials and Methods

Study Design and Setting: A retrospective descriptive study of ED visits was conducted across all state and affiliated private EDs in the Lombardy Region from January 2019 to December 2023. Furthermore, an inferential statistical analysis was performed to compare ED trends between 2019 and 2023. Data from EUOL (“Emergenza Urgenza online”), an emergency and urgency information system implemented in Lombardy, was used for this study. This system serves as a technological platform facilitating information exchange among various components of the emergency system. Each ED in Lombardy automatically submits status updates to the system every three minutes, including details about each ED visit. The central EUOL system collects this information. This study was approved by the AREU (Agenzia Regionale Emergenza Urgenza) Board in accordance with institutional guidelines in line with DGR 315/2023. The data provided by AREU were anonymous, as they do not identify patients and do not represent personal information. Therefore, the study does not require approval from the Ethics Committee or the patients’ informed consent. The year 2023 was selected as the post-pandemic period for analyzing ED visits due to significant shifts in epidemiological trends and public health policy. On 31 March 2022, the Italian government officially ended the state of emergency, initiating a gradual return to normalcy. Following this, on 5 May 2023, the WHO declared the end of COVID-19 as a global health emergency. By 2023, the COVID-19 disease burden, mortality, and hospitalization rates in Italy had markedly declined compared to the pandemic years [28].

Measures and Statistical Analyses: The measures included ED visit volume, patient acuity level, arrival by emergency vehicle, hospital admission rate, and crowding indicators. ED visits were stratified by patient age, sex, arrival by emergency vehicle, triage code, and disposition status upon completion of treatment (admitted, discharged, ED death/death on arrival, left before treatment was complete, refused hospitalization, or transfer to another facility). The patient acuity level was evaluated through the triage code assigned to the patient upon arrival in the ED: red (life-threatening conditions), yellow (potentially life-threatening conditions), green (minor injuries or illness), white (non-urgent conditions), and black (deceased patient). Metrics for ED crowding included the EDLOS, calculated as the monthly and annual time in hours, stratified by discharged patients and admitted patients; door-to-clinician time, calculated as the monthly and annual times in minutes; and left before treatment complete (LBTC) rate, defined as the patients who left without being seen by a physician or against medical advice. The EDLOS of discharged patients measured the time a patient spent in the ED from registration to discharge. The EDLOS of admitted patients measured the time from ED registration to the patient’s transfer to an inpatient unit. The decision to evaluate the EDLOS separately for discharged and admitted patients was made because these groups have distinct patient flow dynamics. For admitted patients, the EDLOS included not only the assessment and treatment phase but also the time spent waiting for an available hospital bed. Door-to-clinician time was defined as the interval between a patient’s registration in the ED and when a clinician saw the patient. For EDLOS calculation, the following outliers were eliminated: times shorter than 10 min or longer than 7 days and any incongruent times where the EDLOS was shorter than the door-to-clinician time. For the door-to-clinician time calculation, times shorter than 0 min or longer than 18 h, as well as patients who left without being seen, were excluded. Count data were expressed as absolute numbers and proportions. Continuous variables were reported as median and interquartile range (IQR). Only complete case analysis was performed, with no imputation for missing data. This study presented an analytical and graphical descriptive analysis. Inferential analysis was conducted solely to compare the years 2019 and 2023. Categorical variables were analyzed using the two-proportion z-test, while continuous variables were compared using the non-parametric Kruskal–Wallis test. All tests were two-tailed, with statistical significance set at *p* < 0.05. Analysis was conducted using R version 3.4.

## 3. Results

### 3.1. Study Sample and ED Visit Volumes

**Figure 1:**
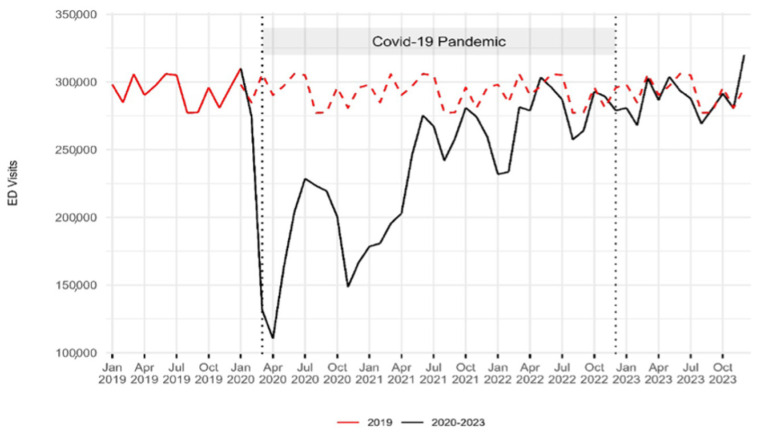
Emergency department visit volume trends: 2019–2023. The red dashed line indicates the 2019 baseline for comparison with the period from 2020 to 2023. Two vertical dashed lines represent the onset of the COVID-19 pandemic in March 2020 and its end in December 2022. A sharp decline in the number of ED visits was observed during the first year of the pandemic, followed by a gradual return to pre-pandemic levels.

**Figure A1:**
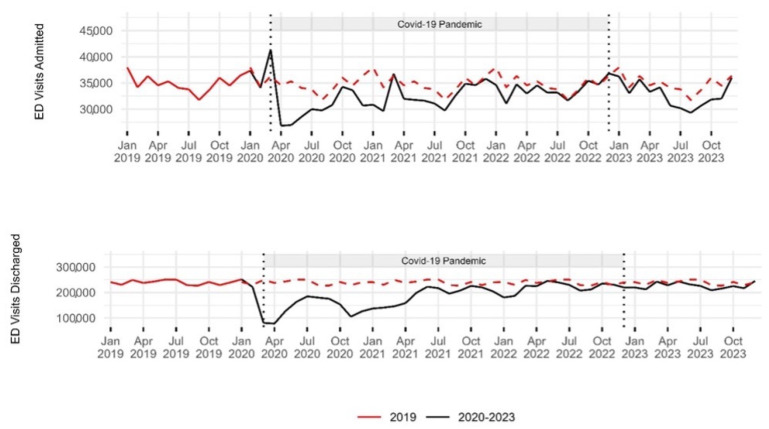
Trends in emergency department visit volume of admitted and discharged patients: 2019–2023. The red dashed line indicates the 2019 baseline for comparison with the period from 2020 to 2023. Two vertical dashed lines represent the onset of the COVID-19 pandemic in March 2020 and its end in December 2022. The number of ED visits ending in hospitalization peaked in March 2020 and then remained below pre-pandemic levels, even in 2023. The number of ED visits ending in discharge showed a notable decrease in the first year of the pandemic, then gradually increased, remaining slightly below pre-pandemic levels in 2023.

The data included 15,515,128 ED visits between 2019 and 2023. With the onset of the COVID-19 pandemic, visits declined across all age groups. In 2020, compared to 2019, visits by patients under 15 fell from 594,592 to 299,904 (−50%); by those aged 15–64 from 1,950,945 to 1,359,697 (−30%); by patients aged 65–80 from 590,415 to 434,288 (−26%); and by those over 80 from 377,519 to 285,396 (−24%). Visit volumes then gradually increased and stabilized by 2023. By that year, visits by patients aged 15–64 reached 99% of 2019 levels, those aged 65–80 reached 96%, and visits by patients under 15 reached 94%. Visits by patients over 80 surpassed 2019 levels, reaching 105% (Figure 1, Table 1). Inferential analysis revealed statistically significant differences in the distribution of age groups between 2019 and 2023, with all *p*-values < 0.01 (Table 1). Regarding the percentage distribution of discharged and admitted patients relative to total ED visits, the proportion of discharged patients declined from 81.6% in 2019 to 77.6% in 2020, while admitted patients increased from 11.9% to 16.2%. By 2023, the percentage of discharged patients reached 78.4%, while admitted patients accounted for 11.4%, with statistically significant variations observed between 2019 and 2023 (*p* < 0.01) (Table 1, Figure A1).

### 3.2. Acuity Level

**Figure A2:**
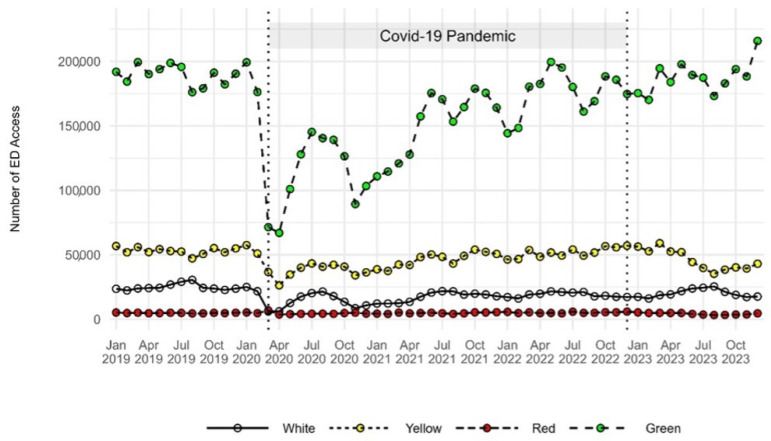
Trends in emergency department visit volume by triage codes: 2019–2023. Two vertical dashed lines represent the onset of the COVID-19 pandemic in March 2020 and its end in December 2022. A notable decline in all triage codes can be observed in March and April 2020. Visits with a green code showed a progressive increase, reaching the highest levels in December 2023.

In absolute terms, in 2020, compared to 2019, visits categorized as white decreased from 348,156 to 205,605 (−41%), green visits from 2,413,238 to 1,572,775 (−35%), yellow visits from 667,713 to 512,502 (−23%), and red visits from 65,927 to 65,092 (−1%) (Table 1, Figure A2). Since the absolute decrease in white and green codes was more pronounced than that in yellow and red codes, the proportion of yellow and red codes increased. Yellow codes rose from 19% to 21.5%, while red codes increased from 1.9% to 2.7%. In 2021 and 2022, there was both an absolute and a proportional increase in red codes, compared to 2019. When comparing 2023 to 2019, white codes decreased from 9.9% (377,519 visits) to 9.0% (310,209 visits), and green codes increased from 68.7% (2,413,238 visits) to 71.7% (2,484,137 visits). Yellow codes fell from 19.0% (667,713 visits) to 17.3% (598,032 visits), and red codes decreased from 1.9% (65,927 visits) to 1.8% (61,253 visits). Statistical analysis revealed significant differences in these proportions between 2019 and 2023 (*p* < 0.01) (Table 1).

### 3.3. Arrival by Emergency Vehicle

In 2019, 21.2% of patients (746,020) arrived at the ED by emergency vehicle. In 2020, this percentage increased to 27.8% (661,520), then gradually decreased to 23.8% (825,014) by 2023. Statistical analysis showed significant variations observed between 2019 and 2023 (*p* < 0.01) (Table 1).

### 3.4. Hospital Admission Rate

**Figure 2:**
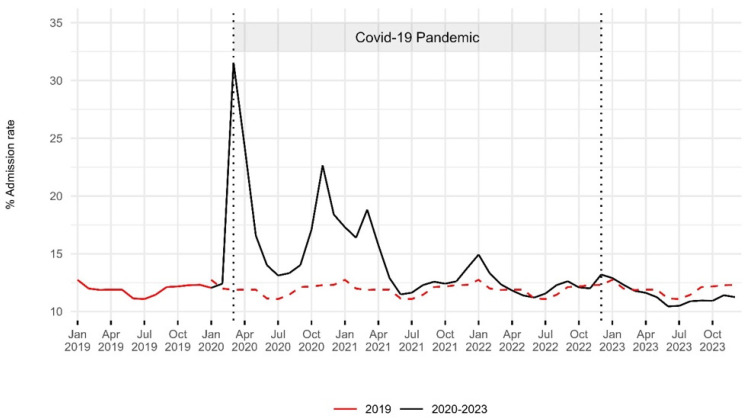
Hospital admission rates in the emergency department: 2019–2023. The red dashed line indicates the 2019 baseline for comparison with the period from 2020 to 2023. Two vertical dashed lines represent the onset of the COVID-19 pandemic in March 2020 and its end in December 2022. An increase in the hospital admission rate was observed during the first two years of the pandemic, with levels in 2023 remaining slightly below pre-pandemic values.

The admission rate peaked at 31.6% (41,429 patients) in March 2020. From March 2020 to March 2022, it remained higher than the 2019 levels. From April to December 2023, it was slightly lower than the 2019 levels (Figure 2). In 2019, admitted patients accounted for 11.9% of the total ED visits (418,427 patients). In 2020, this percentage increased to 16.2% (384,278 patients), followed by a gradual decrease to 11.4% in 2023 (393,139). Statistical analysis revealed significant differences in these proportions between 2019 and 2023 (*p* < 0.01) (Table 1).

### 3.5. ED Crowding Metrics

To calculate the EDLOS of admitted patients, 6395 outliers were removed (0.3% of total admitted patients), while 14,312 outliers were excluded to calculate the EDLOS of discharged patients (0.1% of total discharged patients). To calculate the door-to-clinician time, 681,257 outliers (4% of all ED visits) were removed. This included 585,415 patients who left without being seen.

#### 3.5.1. Median ED Length of Stay

**Figure 3:**
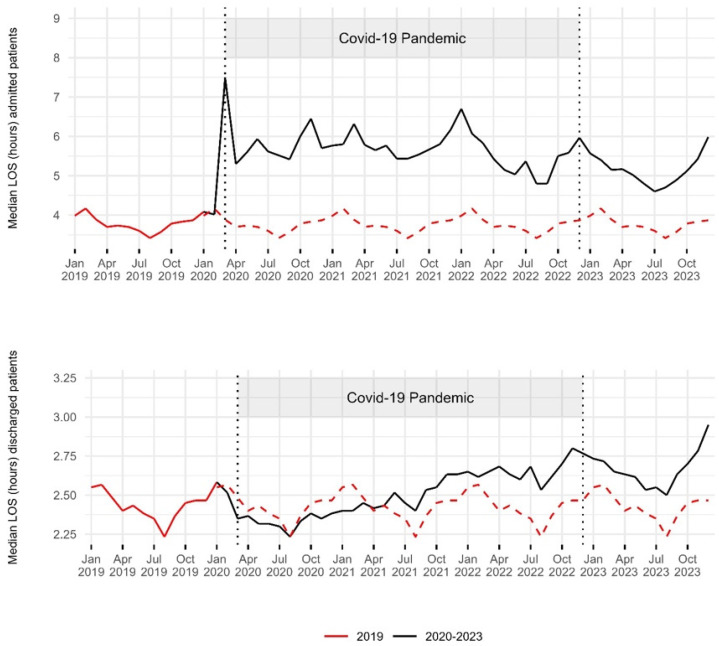
Trends in median ED length of stay of admitted and discharged patients: 2019–2023. The red dashed line indicates the 2019 baseline for comparison with the period from 2020 to 2023. Two vertical dashed lines represent the onset of the COVID-19 pandemic in March 2020 and its end in December 2022. The median EDLOS of admitted patients increased notably since the beginning of the pandemic, remaining above pre-pandemic values throughout 2023. The median EDLOS of discharged patients decreased during the first year of the pandemic, then remained above pre-pandemic values, even in 2023.

**Figure A3:**
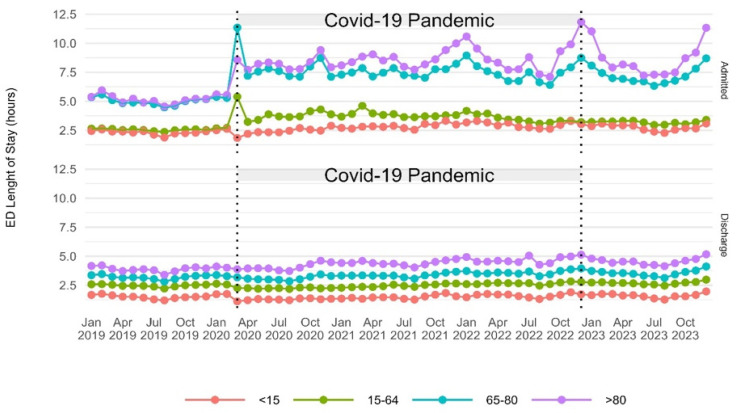
Trends in median emergency department length of stay by age of admitted and discharged patients: 2019–2023. Two vertical dashed lines represent the onset of the COVID-19 pandemic in March 2020 and its end in December 2022. The EDLOS increased with age in both groups. The EDLOS of admitted patients over 65 increased notably, compared to those under 65.

In 2019, the median EDLOS of admitted patients was 3.8 h (IQR [1.6–8.6]). It peaked in March 2020 at 7.5 h. Throughout the pandemic, most values were above 5 h. In 2021, the median EDLOS was 5.8 h (IQR [2.4–16.6]). In 2023, there was a decrease compared to the pandemic period, with a median of 5.2 h (IQR [2.1–17.4]), yet it was still 36% higher than in 2019. Statistical analysis revealed a significant difference between 2019 and 2023 (*p* < 0.01). In 2019, 26.4% of admitted patients experienced an EDLOS exceeding 8 h. This percentage increased during the pandemic period, rising to 38.3% in 2020 and further reaching 39.1% in 2021. The trend remained consistent in 2022 with 38.7% before slightly declining to 37.4% in 2023 (Table 2, Figure 3). In 2023, compared to 2019, the EDLOS of admitted patients increased across all age groups. It was 67% higher for those over 80 years old, 43% higher for those between 65 and 80 years old, 25% higher for those between 15 and 64 years old, and 20% higher for those under 15 years old (Figure A3). The median EDLOS of discharged patients in 2019 was 2.4 h (IQR [1.3–4.4]). From the beginning of the pandemic until March 2021, the monthly medians were lower than in 2019. However, from June 2021, the values gradually increased, reaching their peak in December 2023. In 2023, the median was 2.7 h (IQR [1.4–4.9]), which was 13% higher than in 2019. Statistical analysis showed significant differences between 2019 and 2023 (*p* < 0.01). In 2019, 8.8% of discharged patients had an EDLOS greater than 8 h. This percentage rose to 10.3% in 2020 and increased further to 10.5% in 2021. The trend continued in 2022, reaching 11.4% before slightly declining to 11.2% in 2023 (Table 2, Figure 3). In 2023, compared to 2019, the EDLOS of discharged patients was 16% higher for patients over 80, 11% higher for those aged 65–80, 9% higher for those aged 15–64, and 8% higher for those under 15 (Figure A3).

#### 3.5.2. Door-to-Clinician Time

**Figure A4:**
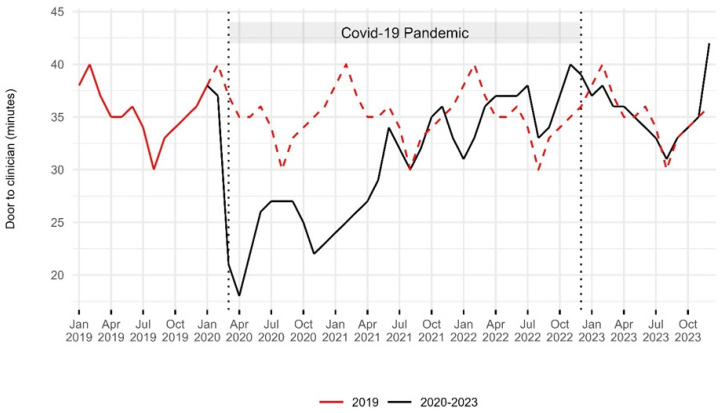
Trends in median door-to-clinician time: 2019–2023. The red dashed line indicates the 2019 baseline for comparison with the period from 2020 to 2023. Two vertical dashed lines represent the onset of the COVID-19 pandemic in March 2020 and its end in December 2022. The median door-to-clinician time decreased during the first year of the pandemic, then gradually returned to pre-pandemic levels in 2023, with an increase observed in December 2023.

The median door-to-clinician time was 35 min (IQR [12–94]) in 2019. It decreased in 2020 and 2021. In 2023, the median was 35 min (IQR [11–97]). Statistical analysis showed significant changes between 2019 and 2023 (*p* < 0.01) (Table 2, Figure A4).

#### 3.5.3. Rate of Patients Leaving Before Treatment Completion

**Figure 4:**
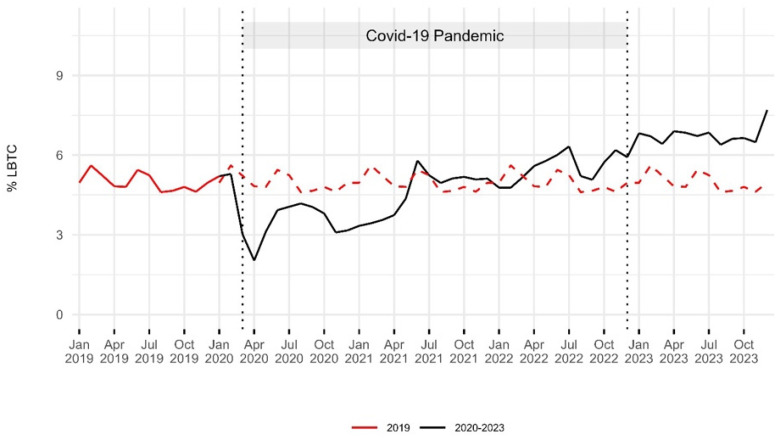
Trends in the rates of leaving the emergency department before treatment completion: 2019–2023. The red dashed line indicates the 2019 baseline for comparison with the period from 2020 to 2023. Two vertical dashed lines represent the onset of the COVID-19 pandemic in March 2020 and its end in December 2022. Rates of LBTC decreased during the first year of the pandemic, then gradually increased, reaching the highest levels in 2023.

The LBTC rate was 5% in 2019 (175,238 patients). It decreased to 4% by 2020 (94,955 patients) but then rose, reaching a peak of 6.8% in 2023 (234,476 patients). Statistical analysis showed significant changes between 2019 and 2023 (*p* < 0.01) (Table 2, Figure 4).

## 4. Discussion

In Lombardy, based on reports from the Italian National Health Service (INHS), the rates of visits to the ED per 1000 members of the population remained relatively stable in the decade before the pandemic [29]. The first Italian community outbreak of COVID-19 was identified on 21 February 2020, with Lombardy being the most affected region. A nationwide lockdown was imposed on 9 March 2020. ED visits dropped and remained lower than in 2019, particularly during the first two years of the pandemic. Visits declined across all age groups, but the smallest reduction was observed among individuals aged 65 and older. This was likely due to their higher burden of multimorbidity and increased incidence of COVID-19, which made them more prone to requiring ED visits and hospitalization [30]. According to the literature, the decrease in ED visits during the pandemic was attributed to various factors. Firstly, the pervasive fear of contracting COVID-19 led many patients to hesitate in seeking emergency care, even when it could be vital [23,24,27]. The decrease in exposure to other transmissible infections during lockdowns and fewer accidents and traumatic injuries resulting from reduced traffic and workplace activities also contributed to this trend [25,31]. During the pandemic, the severity of clinical presentations increased, as evidenced by the rise in the proportion of yellow and red codes. In absolute terms, red code cases increased in 2021 and 2022 compared to 2019, while white and green code cases decreased. The hospital admission rate increased, reaching its peak in March 2020. Similar trends in ED visits were observed in various regions of Italy and countries such as the USA, the UK, and Germany [1,19,21,22,32]. In Lombardy, there was an increase in the proportion of patients arriving at the ED by emergency vehicles. This phenomenon could be attributed to the greater severity of clinical presentations and shifts in patient behavior. During lockdowns, limited public transport may have led patients to rely more on calling ambulances for healthcare needs. Additionally, older individuals, who are more likely to use ambulances for transport, experienced smaller decreases in visit volume [22]. By 2023, ED visits nearly reached 2019 levels. There were 20,660 more ED visits from individuals over 80 years old, compared to 2019. However, this coincided with an increase in the population of residents over 80 in Lombardy. In 2023, patients with low-acuity codes (white and green) accounted for 80.7% of the total, an increase of 2 percentage points, compared to 2019. Research suggests that patients may elect to visit the ED for low-acuity concerns due to a perceived urgency, trust in the hospital, convenient location, faster access to a physician and diagnostic resources, recommendations from other doctors, and the absence of a regular primary care provider [33]. Limited access to timely general practitioner (GP) appointments often drives patients to seek emergency care [7,34]. A Canadian study suggests that improving access to primary care physicians could reduce low-acuity ED visits by approximately 43% [35]. Lombardy has been struggling with a significant shortage of GPs and pediatricians, a situation worsened by the pandemic, with an estimated 1326 additional doctors needed to meet demand in 2023 [36]. To reduce low-acuity visits, in addition to expanding primary care access, it is essential to explore the barriers and motivators influencing patients’ decisions to seek care and enhance education on appropriate ED use [33]. Furthermore, implementing integrated care for people with chronic conditions could help decrease reliance on ED [37]. By 2023, 11.4% of ED patients required hospitalization. In absolute terms, there was a decrease of 25,288 hospitalized patients, compared to 2019. The EDLOS of discharged patients, after a modest decrease in the early months of the pandemic, gradually rose, reaching its highest levels in 2023. The EDLOS for admitted patients increased markedly, particularly among older individuals (65+), with high values persisting into 2023. In 2019, 26.4% of admitted patients had an EDLOS greater than 8 h; by 2023, this had risen to 37.4%. These findings align with other studies showing worsening crowding, despite a decline in visits [1,32,38,39,40,41]. The LBTC rate peaked in 2023, with some evidence suggesting a link between leaving without being seen and ED crowding [42]. During the pandemic, factors related to throughput led to an increase in the EDLOS for all patients. The higher severity of clinical cases, the need for frequent monitoring and ventilatory support, patients’ inability to be autonomous—coupled with the absence of family and caregiver support—and the constant use of personal protective equipment all increased the workload on healthcare staff. Managing two separate patient flows—COVID-positive and COVID-negative—and frequent sanitization also contributed to this rise [43]. Furthermore, the notable increase in the EDLOS of admitted patients can be attributed to the phenomenon of boarding, where admitted patients are held in the ED while waiting for an available inpatient bed. During the pandemic, this was driven by unprecedented demand and the rapid saturation of beds in medium- and high-intensity care units [3,43]. Most admissions were for patients with medical conditions (rather than surgical ones), with a shortage of specialists. Beds from other specialties had to be converted to general medicine, which required time. Additionally, COVID-19 patients had longer stays than surgical or low-acuity patients, which reduced bed turnover and limited availability for new admissions from the ED. Although this study did not directly measure boarding, it is well-recognized in Lombardy’s EDs as a key contributor to increased EDLOS and ED crowding [44]. One reason for the persistence of the high EDLOS among admitted patients during the post-pandemic period could be the reduced availability of hospital ward beds. In 2022, the number of acute care beds in Lombardy decreased, compared to 2019. Additionally, the inpatient length of stay (IPLOS) increased. In 2019, the INHS reported a national IPLOS of 10.2 days for internal medicine wards and 11.4 days for geriatrics wards, which increased to 11.5 and 12.7 days, respectively, in 2022 [29]. According to an Italian survey, half of the hospitalizations in internal medicine wards involve patients over 70 years old, with over 50% staying a week longer than necessary due to a lack of family support and insufficient pensions for nursing home fees. Furthermore, intermediate healthcare facilities are often unavailable, and activating integrated home care is complex [45]. An occupancy rate of about 85% is typically considered the maximum to reduce the risk of bed shortages [46]. In 2022, wards in public hospitals in Italy, such as internal medicine, geriatrics, cardiology, neurology, oncology, and pulmonology, had occupancy rates exceeding 88% [29]. Although we do not have data for 2023, we can hypothesize that these trends persisted or worsened throughout the year. Lastly, staff shortages in ED may also be contributing to the increase in the EDLOS [47]. Emergency medicine faces a significant challenge in Italy, with low interest among doctors in specializing in this field. Stressful and dangerous working conditions, both physically and legally, and limited opportunities for private practice are some of the reasons driving this issue [44].

### Limitations

Some potential biases, such as data loss from system interruptions, manual entry errors, and anomalies like unclosed patient files, may have influenced the results, although excluding outliers could help minimize their impact. The lack of detailed data on healthcare staff in EDs and their variation throughout the analyzed period made it difficult to assess their impact on crowding metrics. Additionally, the absence of data on hospital bed availability, IPLOS, and occupancy for 2023 limited our ability to fully evaluate the study period. We also lacked data on occupancy rates and the IPLOS in Lombardy throughout the study period. Although boarding is a well-recognized factor contributing to the increased EDLOS of admitted patients in Lombardy, its impact could not be assessed in this study due to missing admission decision times. However, improvements in compiling this variable within EDs may enable a more accurate evaluation of boarding in the future.

## 5. Conclusions

In 2023, ED visits in Lombardy increased compared to the pandemic period but remained below 2019 levels. The proportion of high-acuity codes and hospital admissions was slightly lower than in 2019. The rise in median EDLOS and LBTC rates suggests a systemic issue beyond the ED, likely driven by reduced acute care bed capacity, staffing shortages, and inadequate territorial healthcare responses, all exacerbated by the pandemic. A comprehensive approach that improves hospital bed management and availability, facilitates the timely discharge of complex patients through better access to intermediate care facilities and home care services, expands primary care access, and addresses workforce shortages will be crucial for building a more efficient, sustainable, and resilient healthcare system, one that is well-prepared to tackle potential future emergencies.

## Figures and Tables

**Table 1:** Characteristics of ED visits by year in Lombardy. The sample included 15,515,128 ED visits.

	2019		2020		2021		2022		2023		*p*-Value ^†^
Total ED visits	3,514,426		2,380,005		2,861,473		3,294,468		3,464,756		
Patient age (year)											
<15	594,592	16.92%	299,904	12.60%	398,977	13.94%	528,408	16.04%	559,608	16.15%	<0.01
15–64	1,950,945	55.51%	1,359,697	57.13%	1,648,242	57.60%	1,844,961	56.00%	1,935,642	55.87%	<0.01
65–80	590,415	16.80%	434,288	18.25%	485,208	16.96%	536,531	16.29%	566,787	16.36%	<0.01
> 80	377,519	10.74%	285,396	11.99%	328,128	11.47%	383,185	11.63%	398,179	11.49%	<0.01
NA	955	0.03%	720	0.03%	918	0.03%	1383	0.04%	4540	0.13%	<0.01
Sex											
Female	1,779,067	50.62%	1,184,902	49.79%	1,429,372	49.95%	1,631,294	49.52%	1,715,285	49.51%	<0.01
Male	1,734,917	49.37%	1,194,770	50.20%	1,431,690	50.03%	1,662,705	50.47%	1,749,046	50.48%	<0.01
NA	442	0.01%	333	0.01%	411	0.01%	469	0.01%	425	0.01%	0.71
Triage Scale											
White	348,156	9.91%	205,605	8.64%	239,210	8.36%	268,243	8.14%	310,209	8.95%	<0.01
Green	2,413,238	68.67%	1,572,775	66.08%	1,930,559	67.47%	2,264,322	68.73%	2,484,137	71.70%	<0.01
Yellow	667,713	19.00%	512,502	21.53%	590,752	20.65%	659,367	20.01%	598,032	17.26%	<0.01
Red	65,927	1.88%	65,092	2.73%	67,046	2.34%	73,177	2.22%	61,253	1.77%	<0.01
Black	275	0.01%	295	0.01%	257	0.01%	236	0.01%	105	0.00%	<0.01
NA	19,117	0.54%	23,736	1.00%	33,649	1.18%	29,123	0.88%	11,020	0.32%	<0.01
Disposition											
Admitted	418,427	11.91%	384,278	16.15%	391,207	13.67%	406,202	12.33%	393,139	11.35%	<0.01
Discharge	2,869,009	81.64%	1,846,172	77.57%	2,274,918	79.50%	2,640,042	80.14%	2,716,720	78.41%	<0.01
ED Death	5715	0.16%	8773	0.37%	7695	0.27%	8428	0.26%	7309	0.21%	<0.01
LBTC	175,238	4.99%	94,955	3.99%	134,409	4.70%	183,783	5.58%	234,476	6.77%	<0.01
Refuse Hospitalization	20,913	0.60%	15,940	0.67%	18,982	0.66%	21,293	0.65%	21,466	0.62%	<0.01
Transfer	25,124	0.71%	29,887	1.26%	34,262	1.20%	34,720	1.05%	27,751	0.80%	<0.01
Arrival by emergency vehicle	746,020	21.23%	661,520	27.79%	731,018	25.55%	820,734	24.91%	825,014	23.81%	<0.01

^†^ Inferential analysis was conducted solely to compare the years 2019 and 2023. Source: EUOL (“Emergenza Urgenza online”). NA, not available. ED, emergency department. LBTC, left before treatment complete.

**Table 2:** COVID-19 and emergency department crowding metrics: 2019–2023.

	2019		2020		2021		2022		2023		*p*-Value ^†^
Total ED visits	3,514,426		2,380,005		2,861,473		3,294,468		3,464,756		
Crowding Measures											
LBTC (%)	175,238	(5.0%)	94,955	(4.0%)	134,409	(4.7%)	183,783	(5.6%)	234,476	(6.8%)	<0.01
Median EDLOS of admitted patients in hours [IQR]	3.8	[1.6–8.6]	5.5	[2.1–16.3]	5.8	[2.4–16.6]	5.5	[2.3–17.5]	5.2	[2.1–17.4]	<0.01
Median EDLOS of discharged patients in hours [IQR]	2.4	[1.3–4.4]	2.4	[1.2–4.5]	2.5	[1.3- 4.7]	2.7	[1.4–4.9]	2.7	[1.4–4.9]	<0.01
N° of admitted patients with an EDLOS greater than 8 h (%)	110,411	(26.4%)	147,049	(38.3%)	152,769	(39.1%)	157,278	(38.7%)	147,189	(37.4%)	<0.01
N° of discharged patients with an EDLOS greater than 8 h (%)	252,404	(8.8%)	189,943	(10.3%)	239,929	(10.5%)	301,454	(11.4%)	304,565	(11.2%)	<0.01
Median door-to-clinician time in minutes [IQR]	35	[12–94]	27	[10–73]	30	[11–81]	36	[12–96]	35	[11–97]	<0.01

^†^ Inferential analysis was conducted solely to compare the years 2019 and 2023. ED, emergency department; LBTC, left before treatment complete; EDLOS, ED length of stay; N°, number; IQR, interquartile range.

## Data Availability

The data presented in this study are available on request from the corresponding author.
